# Metformin: Possible Use of a Diabetes Drug in Treatment of Cancer

**Published:** 2018

**Authors:** Albert Vacheron, Anne Wynn, Jeff Zuber, Solomon S. Solomon

**Affiliations:** Department of Medicine and Preventative Medicine, Research and Medical Service VAMC Memphis and Division of Endocrinology, University of Tennessee Health Science Center, Memphis, Tennessee, USA

**Keywords:** AMP kinase, antineoplastic, cancer, insulin resistance, mechanism of action, metformin, Type 2 diabetes

## Abstract

The biguanide drug metformin used for treating Type 2 diabetes has anticancer properties and affects many pathways involving glucose metabolism, energy balance, and cell survival. A number of retrospective clinical studies have indicated a reduced risk of cancer and improved cancer outcomes in Type 2 diabetic patients taking metformin. Several of its effects are mediated through the induction of cellular stress and subsequent activation of AMP kinase, but many other mechanisms act independently of AMP kinase activation. Metformin has been shown to inhibit the effects of tumor necrosis factor (TNF)-alpha. TNF-alpha interferes with insulin signaling to produce insulin resistance in the insulin signaling pathway and promotes apoptosis through NF-KB in the apoptosis pathway. In addition, metformin reduces cellular proliferation by decreasing the amount of available insulin or by directly affecting the mammalian target of rapamycin complex involved with regulating protein synthesis. It can prevent tumors from acquiring stem cell-like properties, upregulate apoptotic pathways, and bolster the immune system’s fight against cancer. Gaining a greater understanding of metformin’s various mechanisms of action will continue to elucidate metformin’s role as an effective treatment for cancer.

Since the introduction of biguanides in the 1950s, metformin has become one of the most commonly used drugs to treat hyperglycemia in Type 2 diabetes mellitus (T2DM)patients. It primarily works by increasing insulin receptor sensitivity, increasing cellular glucose uptake, and decreasing hepatic glucose production.^[1]^ In reducing hepatic glucose production, which in T2DM leads to lower fasting plasma glucose concentrations, it is widely thought to indirectly activate AMP-protein kinase (AMPK) through inhibition of Complex I of the electron transport chain in the mitochondria which increases the intracellular AMP: ATP ratio.^[2]^ Metformin acts mainly through AMPK in downregulating expression of several gluconeogenic genes and affects lipid metabolism leading to decreased steatosis. Although AMPK is thought to be the preferred mechanism for metformin action, other AMPK independent mechanisms impacting glucose and lipid metabolism have also been demonstrated.^[3–5]^

In 2005, an epidemiological study proposed a connection between T2DM patients’ use of metformin as an antidiabetic medication and a reduced risk of cancer.^[6]^ More recent retrospective studies have shown increased overall survival rates, fewer recurrences, and longer periods of remission in T2DM patients suffering from colon, lung, gastroesophageal, thyroid, and prostate cancers.^[7–9]^ Given metformin’s impact on insulin signaling and glucose and lipid metabolism, it is of great interest to researchers to investigate the possibility of repurposing an old T2DM drug into a potential anticancer adjuvant. This review examines a few of the molecular targets and pathways that may play a role in metformin’s antineoplastic mechanisms of action.

Insulin has been shown to promote cancer growth by increasing glucose uptake and utilization through the phosphatidylinositol-3-kinase (PI3K) pathway and cell proliferation by mitogen-activated protein kinase activation.^[10]^ Insulin is a mild growth factor regulating cell division and inhibiting apoptosis, but its real risk for promoting cancer is its ability to potentiate the mitogenic actions of other growth factors. Insulin can activate farnesyltransferase to farnesylate RAS to send it to the plasma membrane and prime it for activation by other growth factors. In the context of hyperinsulinemia, the excessive activation of farnesyltransferase leading to more membrane attached RAS enhances the mitogenicity of insulin and other growth factors.^[11]^ Too much insulin also causes insulin-like growth factor 1 (IGF-1) to be displaced from its binding proteins and decreases IGF binding protein production. IGF-1 is a more powerful growth factor than insulin that can also bind to insulin receptors and can stimulate the RAS-RAF-MEK-ERK or the PI3K/AKT/mammalian target of rapamycin (mTOR) pathways while also inhibiting the antiapoptotic phosphatase and tensin homolog (PTEN).^[12]^ A study examining the role of tumor necrosis factor (TNF)-alpha in insulin resistance demonstrated that the insulin signaling and apoptosis pathways are intimately connected through PTEN phosphatase, TNF-alpha, and insulin.^[13]^ PTEN inhibits PI3K signaling of the insulin pathway producing insulin resistance. In contrast, PTEN, stimulated by TNF-alpha, promotes apoptosis through its actions on NF-KB.^[14,15]^ In addition to decreasing circulating insulin levels through increased insulin receptor sensitivity, metformin has been shown to decrease IGF-1 receptor and insulin receptor expression in endometrial cancer cell lines.^[16]^ The lower levels of circulating insulin and IGF-1 reduce the amount of binding to the insulin receptor, especially to isoform A in cancer cells, leading to less tumor growth.^[17]^

While having an indirect effect by lowering insulin levels, much of metformin’s other antineoplastic activity has been attributed to AMPK activation. AMPK has been shown to inhibit mTOR which regulates protein synthesis and cell proliferation.^[18]^ AMPK phosphorylates tuberous sclerosis complex 2 leading to an inhibition of mTOR in combination with tuberous sclerosis complex 1.^[19]^ AMPK can also directly phosphorylate raptor, a regulatory protein associated with the mTOR complex, to prevent mTOR activation.^[20]^ Metformin also inhibits Rag GTPases, a family of proteins usually sensitive to amino acid levels, which are needed to translocate mTOR to the nucleus so it can complex with RAS-homolog enriched in brain to be activated. Similar to amino acid withdrawal, metformin induces a cytosolic withdrawal and inactivation of mTOR from the nucleus.^[21]^ Another group using phosphoproteomics have found that certain mTOR substrates are still positively regulated in cancer cells following metformin treatment and proposed that metformin does not inhibit mTOR but rather rewires its signaling. In their study, metformin-treated cancer cells were less sensitive to growth following IGF-1 stimulation and displayed increased activity in PP2A phosphatase which deactivates p70S6K, a kinase downstream of mTOR signaling.^[22]^ In pancreatic cancer cell lines, potential crosstalk exists between the insulin/IGF-1 stimulated PI3K/AKT/mTOR pathway and the G_q_PCR stimulated mTOR pathway. Addition of insulin potentiates phosphatidylinositol 4,5-bisphosphate hydrolysis and increases intracellular Ca2+ concentration activated by G_q_PCR agonists such as bradykinin and angiotensin II. This occurred in a mTOR dependent manner as treatment with rapamycin negated the potentiating effects of the insulin. Administration of metformin reversed the insulin-induced Ca2+ mobilization following the addition of G_q_PCR agonists leading to a decrease in DNA synthesis.^[23]^

In many cancers, reprogramming of metabolic pathways occurs to support rapid tumor growth and cellular immortality. In pancreatic ductal adenocarcinoma, the KRAS oncogene is responsible for >90% of the cases.^[24]^ A population of pancreatic cells survives after ablation of the KRAS oncogene, and they retain tumorigenic potential. These surviving cells are termed cancer stem cells (CSCs) and tend to be resistant to conventional chemotherapy due to their slower growth rate and a number of anti-apoptotic mechanisms.^[25]^ Hypoxia-inducible factor 1-alpha (HIF1-alpha), activated in a hypoxic tumor microenvironment, drives expression of pluripotent genes Oct4, Sox3, and KLF-4 which helps influence the phenotype of CSCs.^[26]^ Metformin activated AMPK inhibits HIF1-alpha which negatively impacts the Warburg effect and suppresses tumor growth due to decreased aerobic glycolysis and downregulation of Oct4.^[26,27]^ The Warburg effect is a phenomenon in which cancer cells preferentially use aerobic glycolysis which yields one mole of ATP per mole of glucose compared to 38 moles of ATP per mole of glucose in oxidative phosphorylation through the citric acid cycle.^[27]^ These CSCs have higher oxygen consumption and higher expression of voltage-dependent anion channel (VDAC), a mitochondrial marker, and addition of oligomycin, a mitochondrial stressor, did not increase their rate of glycolysis. The CSCs’ lack of compensation to a mitochondrial stressor may indicate that a compound targeting oxidative phosphorylation (such as metformin in a diabetic patient) in combination with targeted treatment for an oncogenic pathway may be a more effective therapy to eliminate pancreatic tumors and prevent their relapse.^[25]^

Metformin has been shown to affect apoptosis and encourage cell cycle arrest. Activated AMPK phosphorylates MDMX, a protein that regulates P53 ubiquitination and turnover, leading to its stabilization and activation in promoting apoptosis or cellular senescence.^[28]^ Cyclin D1 is overexpressed in many cancers including cancers of the breast, esophagus, lung, and bladder due to a defect in post-translational regulation. Hence, drugs that impact Cyclin D1 expression are of great therapeutic interest.^[29]^ Metformin has been shown in three prostate cancer cell lines to block cell cycle progression from G0/G1 to S as seen by a decrease in Cyclin D1, E2F1, and Rb phosphorylation. It is known that phosphorylation of Cyclin D1 at Thr286 induces its degradation in the proteasome, but it is unclear if metformin leads to its phosphorylation.^[30]^ On the transcriptional level, protein regulated in development and DNA damage response 1 (REDD1) expression is increased after metformin administration. Metformin works through p53 to increase REDD1 expression and decrease Cyclin D1 expression. The net effect is to induce cell cycle arrest and apoptosis.^[31]^

In addition to inhibition of Complex I of the electron transport chain, metformin binds directly to Hexokinase II to induce apoptosis. Hexokinase II has been previously shown to be upregulated in cancers and binds to its mitochondrial outer membrane receptor VDAC to phosphorylate glucose using ATP produced in the mitochondria.^[32,33]^ Metformin blocks the Hexokinase II phosphorylating activity uncompetitively after the enzyme-substrate complex is formed and binds in the region that glucose-6-phosphate normally occupies. On metformin binding, Hexokinase II preferentially localizes to the cytosol and does not bind to the outer mitochondrial membrane.^[33]^ Previous studies have shown that Hexokinase II inhibition and disassociation from the mitochondrial outer membrane have a potent anti-tumor effect through increased mitochondrial membrane permeability and release of apoptotic proteins into the cytosol.^[34,35]^ Furthermore, AKT phosphorylates Hexokinase II at Thr473 which enables its association with the mitochondrial membrane to perform its enzymatic function and protect the cell from leakage of apoptotic proteins.^[36]^ Metformin can block AMPK dependent AKT phosphorylation which is normally induced by IGF-1.^[37]^ This additional indirect mechanism of Hexokinase II perturbance further complements metformin’s antineoplastic efficacy as a glucose metabolism disrupter to promote mitochondrial membrane permeability and subsequent apoptosis.

Despite the many beneficial effects of metformin being mediated by AMPK, metformin has been shown to have other potential anticancer mechanisms independent of AMPK activation. Metformin blocks NF-KB preventing transcription of pro-inflammatory cytokines such as TNF-alpha needed to promote tumor growth in prostate cancer cells.^[38]^ It plays a role in the immune system’s fight against cancer by strengthening the memory T cell response following administration of an experimental anticancer vaccine by regulating genes involved in fatty acid oxidation.^[39]^ Furthermore, metformin was shown to increase the efficacy of tumor-infiltrating T lymphocytes by inducing a phenotype switch from a central memory T cell to an effector memory T cell resulting in increased production of tumor-fighting cytokines and decreased T cell apoptosis.^[40]^ Another gene regulatory effect of metformin is activation of p38 to phosphorylate and destabilize specificity protein-1 (SP-1).^[22]^ This downregulates SP-1 targeted genes including pyruvate kinase.^[41]^ Metformin downregulated the Stat3-Bcl2 pathway to inhibit cell growth in esophageal squamous cell carcinoma by increasing autophagy and apoptosis.^[42]^ Finally, metformin’s antineoplastic action can induce RNA degradation of C-myc through increased expression of miR-33a, a miRNA which binds to C-myc RNA following upregulation of the DICER enzyme.^[43]^

The recent revelation of metformin’s ability to reduce cancer risk in T2DM patients has generated even greater interest in its potential impact on both cancer and T2DM. These effects may or may not be primarily regulated by AMP kinase. Given that T2DM patients are already at a greater risk of developing cancer, the ability to adapt one of the most common diabetic drugs to cancer treatment could have tremendous implications clinically.^[44]^ Beyond its direct anticancer effects, some through AMPK, researchers are continuing to discover a myriad of actions related to glucose and lipid metabolism, energy balance, and genomic regulation, all of which may contribute to metformin’s reduction in cancer risk among T2DM patients. Ongoing research will result in a better understanding of metformin’s mechanisms and will hopefully enable physicians to optimize care in concert with other antineoplastic treatments. In the future, we anticipate metformin or a derivative drug may be used in cancer therapy independent of a patient’s diabetic status.
